# Whole blood stimulation provides preliminary evidence of altered immune function following SRC

**DOI:** 10.1186/s12865-023-00595-8

**Published:** 2024-01-13

**Authors:** Alex P. Di Battista, Shawn G. Rhind, Maria Shiu, Michael G. Hutchison

**Affiliations:** 1 Defence Research and Development Canada, Toronto Research Centre, Toronto, ON Canada; 2 Faculty of Kinesiology and Physical Education, University of Toronto, Toronto, ON Canada; 3 David L. MacIntosh Sport Medicine Clinic, Faculty of Kinesiology & Physical Education, University of Toronto, Toronto, ON Canada; 4 Centre for Sport-Related Concussion Research, Innovation, and Knowledge, University of Toronto, Toronto, ON Canada

**Keywords:** Sport concussion, Inflammation, Bayesian modelling, Causal inference, Directed acyclic graph, Immune stimulation, Cytokines

## Abstract

**Purpose:**

To implement an approach combining whole blood immune stimulation and causal modelling to estimate the impact of sport-related concussion (SRC) on immune function.

**Methods:**

A prospective, observational cohort study was conducted on athletes participating across 13 university sports at a single academic institute; blood was drawn from 52 athletes, comprised of 22 athletes (n = 11 male, n = 11 female) within seven days of a physician-diagnosed SRC, and 30 healthy athletes (n = 18 female, n = 12 male) at the beginning of their competitive season. Blood samples were stimulated for 24 h under two conditions: (1) lipopolysaccharide (lps, 100ng/mL) or (2) resiquimod (R848, 1uM) using the TruCulture® system. The concentration of 45 cytokines and chemokines were quantitated in stimulated samples by immunoassay using the highly sensitive targeted Proximity Extension Assays (PEA) on the Olink® biomarker platform. A directed acyclic graph (DAG) was used as a heuristic model to make explicit scientific assumptions regarding the effect of SRC on immune function. A latent factor analysis was used to derive two latent cytokine variables representing immune function in response to LPS and R848 stimulation, respectively. The latent variables were then modelled using student-t regressions to estimate the total causal effect of SRC on immune function.

**Results:**

There was an effect of SRC on immune function in males following SRC, and it varied according to prior concussion history. In males with no history of concussion, those with an acute SRC had lower LPS reactivity compared to healthy athletes with 93% posterior probability (pprob), and lower R848 reactivity with 77% pprob. Conversely, in males with a history of SRC, those with an acute SRC had higher LPS reactivity compared to healthy athletes with 85% pprob and higher R848 reactivity with 82%. In females, irrespective of concussion history, SRC had no effect on LPS reactivity. However, in females with no concussion history, those with an acute SRC had higher R848 reactivity compared to healthy athletes with 86% pprob.

**Conclusion:**

Whole blood stimulation can be used within a causal framework to estimate the effect of SRC on immune function. Preliminary evidence suggests that SRC affects LPS and R848 immunoreactivity, that the effect is stronger in male athletes, and differs based on concussion history. Replication of this study in a larger cohort with a more sophisticated causal model is necessary.

**Supplementary Information:**

The online version contains supplementary material available at 10.1186/s12865-023-00595-8.

## Introduction

There are many challenges impeding progress in our understanding of the immune response following sport-related concussion (SRC). Animal model research has been helpful in hypothesis generation for human studies across the severity spectrum of brain injury from concussion [1] to severe traumatic brain injury (TBI) [2, 3]. However, differences in animal and human immune systems make translation challenging [4–6] and it can be difficult to design human experiments to validate animal findings. Human studies, which have relied almost entirely on the evaluation of cytokines and chemokines measured in the systemic circulation [7–11], have been informative but have not yet gone beyond speculative group differences in individual biomarker concentrations between healthy and injured groups, or the identification of correlations between biomarker levels and clinical outcomes (symptoms, recovery, etc). The complexity of the immune response and its pleiotropic and redundant features make interpretation of these findings difficult; it is not clear how elevated/depressed concentrations of individual blood cytokines relate to immune system function or status. While multiple marker panels evaluated with multivariate statistical models can help identify signatures and infer system-level changes, functional interpretation remains difficult when looking at static measures in the blood at a given time.

It may be advantageous to assess immune function by stressing or challenging the immune system and quantifying the reactivity to a particular stimulus with a known signaling pathway [12–14]. Here, when group differences are estimated, they more closely approximate differences in function of a specific facet of the immune system. For example, prior studies on immune biomarkers in SRC have focused on a broad suite of inflammatory cytokines and chemokines [8–11] that are common products of Nuclear Factor Kappa B (NF-kB) transcription [15, 16]. In the innate immune system, NF-kB-mediated cytokine production is classically linked to toll-like receptor (TLR) signalling [15]. While this is a likely mechanism at play in the acute phase post SRC, there are also several other potential pathways that may be involved, such as the sympathoadrenal-immune response [17] or the inflammasome [18]. Without evaluation of cytokine reactivity to direct stimulation of known pathways, it is difficult to understand the etiology of post-injury immune activity.

In addition to shifting the methodological paradigm of assessing immune function using blood biomarkers, statistical considerations may improve the quality of study results. First, a change in focus to effect estimation as opposed to *p* values and significance testing could improve results interpretation. Arbitrary, historically determined cut points have constrained findings into a false dichotomy of mattering (significant) or not mattering (not significant), which is often incorrect, or at the very least oversimplistic in biological systems [19–23]. Second, causal modelling that provides *a priori* transparency of scientific beliefs would also provide clarity and simplify efforts at replication [22, 24]. Heuristic models like directed acyclic graphs (DAGs) can be useful to advance knowledge and inform future studies because they are explicit in their assumptions and come with a set of simple rules for effect estimation [22, 24]. Indeed, identifying group differences and correlations in data without explicitly expressed scientific beliefs is potentially misleading; confounding and colliding variables can induce false relationships between an exposure and an outcome, mediating/moderating variables erroneously adjusted for can eliminate real effects, and competing causes can hamper precision [22, 24–27].

The application of causal modelling to study the immune system following SRC can draw from one of several lines of research. First, early animal models and human studies on TBI have suggested acute inflammation in response to the injury [1, 2, 28], with speculation of chronic persistence [28–30]. Human SRC data from our group and others has shown that individual inflammatory cytokines and chemokines such as monocyte chemoattractant protein (MCP)-4 and macrophage inflammatory protein (MIP)-1β may be elevated within the first week following injury [8], inflammatory gene expression may be decreased [16], and elevated cytokines have been observed in healthy individuals with a concussion history [10, 31]. Animal model research has also suggested a phenomenon known as ‘microglial priming’ may occur following an initial TBI/concussion [29, 30, 32, 33], possibly leading to an amplified reaction to subsequent injuries and providing a potential pathway to neurodegeneration [29, 32]. Interestingly, we have previously observed an interaction between IL-6 and concussion history in those with an acute SRC [7]. Furthermore, given the noted differences in recovery trajectories in males and females following SRC [34–37], the general difference in male and female immunity [38], and some preliminary work by our group showing potentially contrasting biomarker signatures following injury [9], it seems reasonable that males and females have a different immunological response to SRC. However, and importantly, all the human work, including our own, was done within a null hypothesis significance testing framework that relied upon an arbitrary decision theoretic cut point of *p* < 0.05, without a causal model.

This preliminary study aimed to implement whole blood stimulation within a causal analytical framework to estimate the effect of SRC on immune function. To achieve this, we derived a DAG based on hypotheses generated from prior literature of how SRC and concussion/TBI may alter immunity. Immune function was measured through the stimulation of whole blood *ex-vivo* using common inflammatory ligands LPS and R848, and subsequent quantitation of a multi marker panel of cytokines and chemokines.

## Methods

### Participants

Fifty-two athletes from a Canadian university’s sport program participated in this study during the 2018/2019 academic year; this sub study was part of a larger project conducted by our group from 2013 to 2019. Of the 52-athlete convenience sample, 22 athletes (n = 11 female, n = 11 male) from seven sports were enrolled within a week (median = 4 days, interquartile range [IQR] = 3–5) of being diagnosed with an SRC; 30 healthy athletes (n = 18 female, n = 12 male) from 11 sports were enrolled at the beginning of their competitive season. Concussion diagnosis and medical clearance decisions were made by a staff physician at the university sport medicine clinic in accordance with the Concussion in Sport Group guidelines [39]. Prior to enrollment, all participants were provided written informed consent. All study procedures were in accordance with the declaration of Helsinki, and approved by the Health Science Research Ethics Board, University of Toronto (protocol reference # 27958).

### Blood collection and stimulation

Blood was sampled via standard venipuncture from athletes at the time of study enrolment. Athletes were excluded if they were currently symptomatic from a known infection, illness, or seasonal allergies, or for taking any medications beyond birth control; in the sample used for this study, no athletes were excluded. Blood was drawn via standard venipuncture into 4 ml vacutainers coated with heparin. At this point, heparinized blood was transferred into the TruCulture® system (Rules Based Medicine, Q^2^ Solutions, Texas, USA) for stimulation in two separate tubes containing either the toll like receptor 4 (TLR4) ligand Lipopolysaccharide (LPS, 100 ng/mL) or the TLR7/8 ligand resiquimod (R848, 1 uM). Briefly, 1 ml of blood was pipetted into each of the TruCulture® tubes and placed on a benchtop heatblock (VWR, USA) where they were kept at 37 °C for 24 h. Following stimulation, a plunger was inserted into the tube to separate the cells from the cell supernatant. The supernatant was collected and then stored at -80 °C until analysis.

### Biomarker analysis

Stimulated supernatant samples were analyzed by immunoassay using the protein biomarker platform Olink® (Olink, Uppsala, Sweden). The commercially available ‘Target 48’ cytokine panel was run according to manufacturer’s instructions at a certified clinical research laboratory. Given that the samples were stimulated, a 1:100 dilution was applied before the assays were run. Each stimulated tube was also accompanied by a ‘Null’ control tube without the stimulant present. However, preliminary analyses by our group found that the stimulants used in the current study induced such a substantial level of cytokine production compared to the Null tube (orders of magnitude in most relevant markers) that subtracting the Null cytokine values from the stimulated cytokine values made no difference in the estimates derived from our statistical models. Thus, for simplicity, we only analyzed and reported the results from the stimulated tubes in our sample. The 45 cytokines evaluated using the Target 48 panel can be found at the Olink website using the following link: https://olink.com/content/uploads/2021/09/olink-target-cytokine-48-panel-content-v1.0.pdf.

### Symptoms

Athletes reported their symptoms on the day of the blood draw by completing a 22-item post-concussion symptom scale where questions were answered using a seven-point Likert rating. This symptom questionnaire is part of the Sport Concussion Assessment Tool (SCAT), the most widely used tool to assist in the diagnosis, management, and prognosis of individuals with concussion [40, 41]. A total symptom score was obtained by summing the presence or absence of each symptom irrespective of severity, with a maximum value of 22; symptom severity was evaluated by summing the rated symptom score for each symptom.

### Data analysis

Our aim was to estimate the effect of SRC on immune function in the acute/subacute phase (within 7 days post-injury). Immune function was measured using a panel of cytokines and chemokines commonly associated with inflammation in response to stimulation with two well-characterized inflammatory agents (LPS and R848), which are known to cause the production several cytokines and chemokines through TLR-mediated signalling [39, 40]. The analysis plan consisted of three steps: (1) create a heuristic scientific model in the form of a DAG to make explicit modelling assumptions regarding the effect of SRC on immune function, (2) create two latent cytokine variables representing LPS and R848 reactivity, respectively, and (3) employ the rules of causal inference to estimate the effect of SRC on immune function through student-t regression modelling, with the latent variables created in step 2 serving as proxies of immune function.

### Heuristic directed acyclic graph of concussion and immune function

**Fig. 1:**
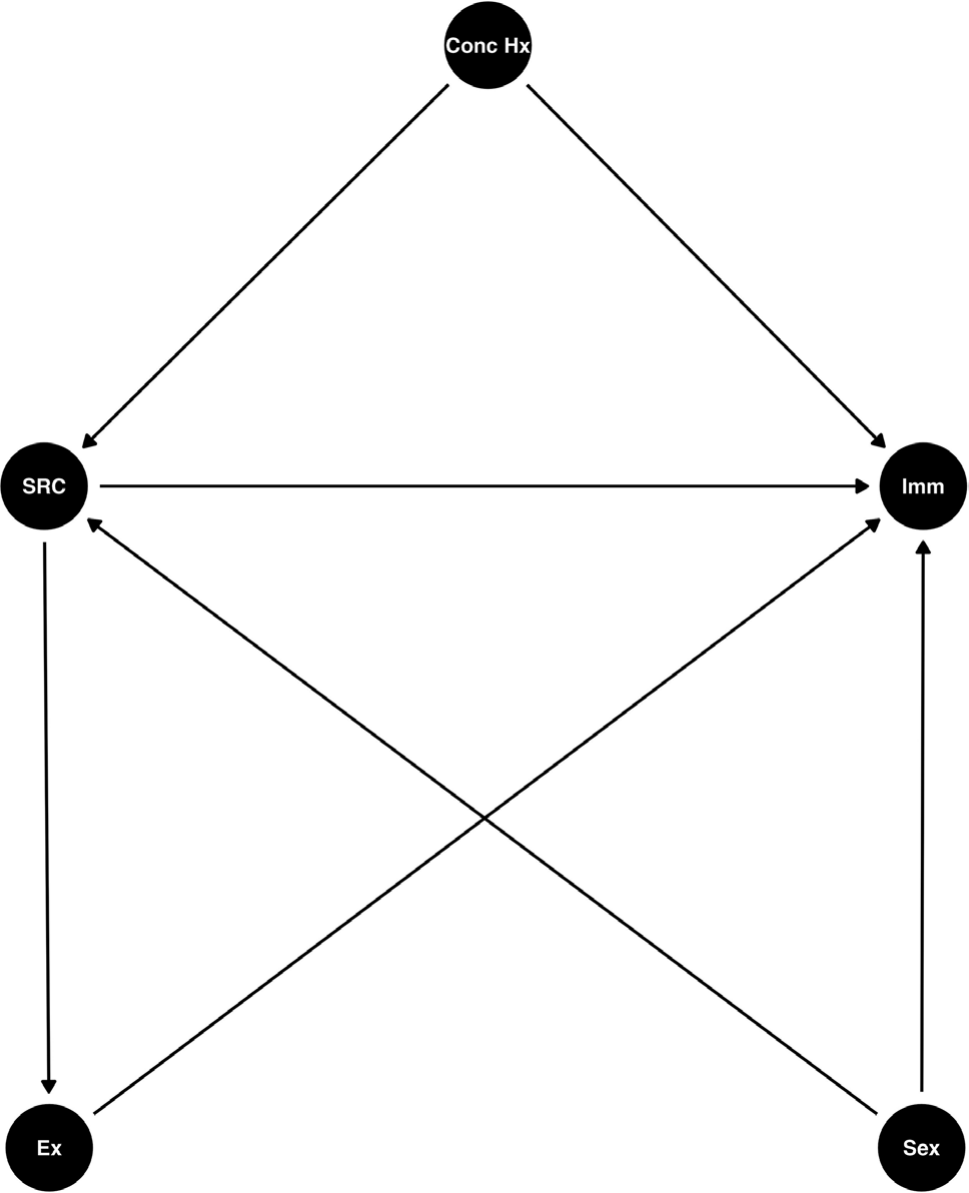
A Directed Acylic Graph of the effect of Sport related concussion on immune function. Conc Hx, Concussion history; Imm, immune function; SRC, sport-related concussion; Ex, current exercise status. A heuristic scientific model in the form of a directed acyclic graph (DAG) used to model the effect of SRC on immune function

To arrive at a generative statistical model, we first used a heuristic DAG (Fig. 1) to map out our scientific beliefs based on our own prior work and that of others. We believed that SRC would influence immune function, and that the effect would be moderated by sex [9]. Given the historical precedent of ‘priming’ [7, 29, 33, 38] we believed that prior concussion history would interact with an acute concussion to influence immune function. There were two backdoors into the SRC node in our DAG because sex and concussion history were not equal across groups in our sample. Furthermore, we acknowledge the possibility that due to the initial period of rest commonly observed following SRC, and given the relationship between acute exercise and inflammation [42, 43], a potential change in exercise behaviour in an active population may alter immune function,. However, as we did not capture the type and time from exercise in our study, this is an unmeasured mediating variable; hence, given the rules of causal inference [24] and the DAG in Fig. 1, we could estimate the total effect of SRC on immune function, but were unable to measure the direct effect.

### Latent modelling of cytokines

LPS and R848 cause the synthesis and release of inflammatory cytokines and chemokines from cells into the systemic circulation in a coordinated fashion. To capture the nature of this process, we employed a Bayesian latent factor model to estimate a single variable comprised of the weighted contributions of each individual cytokine and chemokine in response to either LPS and R848 stimulation. We then used these variables as a proxy of immune function for downstream modelling of the DAG in Fig. 1. For model explanation, including the statistical notation used, please see the Supplementary Material 1: Supplementary Methods. For raw circulating cytokine/chemokine concentrations, please see Supplemental Table 1.

### Missing data

Cytokines and chemokines are often found in low concentrations in the peripheral blood and are frequently below the quantitation range of commercial assays. While stimulation helps alleviate this concern by elevating the blood concentration of several mediators by orders of magnitude, in a large panel of markers there will often be some that either do not respond to stimulation or respond to a lesser degree. Hence, missingness is not completely at random (MCAR) nor is it random (MAR), and therefore requires special consideration [22]. In the present study, for values that were below the quantifiable limits of the assay, we used Bayesian multiple imputation [22] within a confined range between zero and the lowest quantifiable value found in the sample data for each cytokine. This imputation strategy was validated on its ability to recover the latent structure of simulated data. In our simulations, data structure was preserved when several markers were missing up to 50% of their lowest values. For more information on the imputation strategy, please see the simulated data and code at the GitHub link associated with this publication. A table quantifying the missing data for each of the markers used in this study under each condition can also be found in Supplementary Table 2.

### Student-t regression

Student-t regressions [2] were used to estimate the total causal effect of SRC on immune function (y in [2]). According to the rules of causal inference [24] applied to our DAG (Fig. 1), to estimate the total causal effect of SRC on immune function we had to adjust for sex and concussion history. We also interacted these variables, as we believed that concussion history interacts with an acute concussion to modulate the immune response, and we believed that the effect of SRC on immune function differs in males and females (moderating effect). Because the number of SRC participants in our study was low (n = 22) and subclassification of concussion history and sex were needed, data coverage for all model parameters was a concern. Student-t models were chosen in place of linear models due to the adaptive degrees of freedom parameter (ν) which the model can learn to help put the appropriate amount of weight in the tails of the distribution. This served to stabilize model estimates and protect against leverage points [22]. Regularizing priors were used for all parameters, and in the interaction term where data coverage was lowest, adaptive priors were used to allow for information sharing and regularization across all interaction term parameter estimates [22]. As a result, posterior group-level parameter estimates were used to create posterior contrasts to estimate group differences in LPS and R848 reactivity, respectively. All data were z-score transformed prior to modelling. For the notation of the statistical model, please see the Supplementary Material 1: Supplementary Methods.

### Algorithm used to provide estimates

Posterior distributions for all estimates were derived using Hamiltonian Monte Carlo as implemented in Stan through RStan [44, 45] (version 2.21) via R [46] (version 4.3) and the RStudio Integrated Development Environment [47] (version 2023.03.1). The R package ‘rethinking’ [48] was used to aid in the post processing of posterior samples and for the creation of density plotting. Latent factor plots were created using the ‘ggplot2’ [49], and tidybayes [50] packages. Tables were made using the gt [51] and gtsummary [52] packages. Latent models were validated on simulated data, and all models were assessed for convergence by inspection of trace plots, R-hat values, and effective sample sizes. For student-t models, a non-centered parameterization was employed to allow full exploration of the entire parameter space and prevention of divergent transitions. Priors were selected via prior predictive simulation to span a scientifically credible range of outcomes, and to regularize posterior parameter estimates. The prior distributions were included in all results figures for transparency and to show the influence of the sample data on the model.

All models were evaluated for out-of-sample performance and leverage points using Pareto-smoothed importance sampling cross-validation via the ‘loo’ package [53]. Data and code used in this study for latent modelling, student-t modelling, latent model simulations under varying levels of data missingness, model checks, Stan model files, figures, and tables, can found in a public GitHub repository (https://github.com/dibatti5/Di-Battista-et-al-2023-JNI-Whole-blood-stimulation-).

## Results

### Participants

Participant characteristics can be seen in Table 1. Age was similar in both groups (median = 21 years), although there were slightly more females in the healthy group (60% vs. 50% in the SRC group), and more athletes in the healthy group without a history of concussion (60% vs. 45% in the SRC group). In those with a history of concussion, both groups had a median time of ~ 2 years from the time of their last concussion to the time of study enrolment. Athletes with SRC presented with a median total 15 symptoms (IQR = 8–23) and a median symptom severity of 36 (IQR 12–67). The median days to recovery was 37 (IQR 21–71). SRC athlete characteristics can be seen in Table 2.

**Table 1:** Participant characteristics

Characteristic	Healthy, N = 30^*1*^	SRC, N = 22^*1*^
**Age**	21 (19, 23)	21 (20, 22)
**Sex**		
Female	18 (60%)	11 (50%)
Male	12 (40%)	11 (50%)
**Concussion History**		
None	18 (60%)	10 (45%)
One	9 (30%)	7 (32%)
More than one	3 (10%)	5 (23%)
**Years Since Last Concussion**	2 (1, 5)	2 (1, 5)
**Sport**		
Basketball	4 (13%)	0 (0%)
Field Hockey	1 (3.3%)	1 (4.5%)
Figure Skating	0 (0%)	1 (4.5%)
Football	0 (0%)	2 (9.1%)
Ice Hockey	12 (40%)	4 (18%)
Lacrosse	2 (6.7%)	1 (4.5%)
Mountain Biking	0 (0%)	1 (4.5%)
Softball	0 (0%)	1 (4.5%)
Rowing	0 (0%)	1 (4.5%)
Rugby	1 (3.3%)	8 (36%)
Soccer	2 (6.7%)	1 (4.5%)
Swimming	0 (0%)	1 (4.5%)
Volleyball	8 (27%)	0 (0%)

SRC, Sport-related concussion

^*1*^ Median (IQR); n (%)

**Table 2:** SRC characteristics

Characteristic	N = 22^1^
Days From Injury To Blood Draw	4 (3–5)
Total Symptoms	15 (8–23)
Symptom Severity	36 (12–67)
Days to Clinical Recovery	37 (21–71)
Recovery > 30 Days	13 (68)

^1^ Median (IQR); n (%)

SRC, sport-related concussion.

### Latent cytokine modelling

**Fig. 2:**
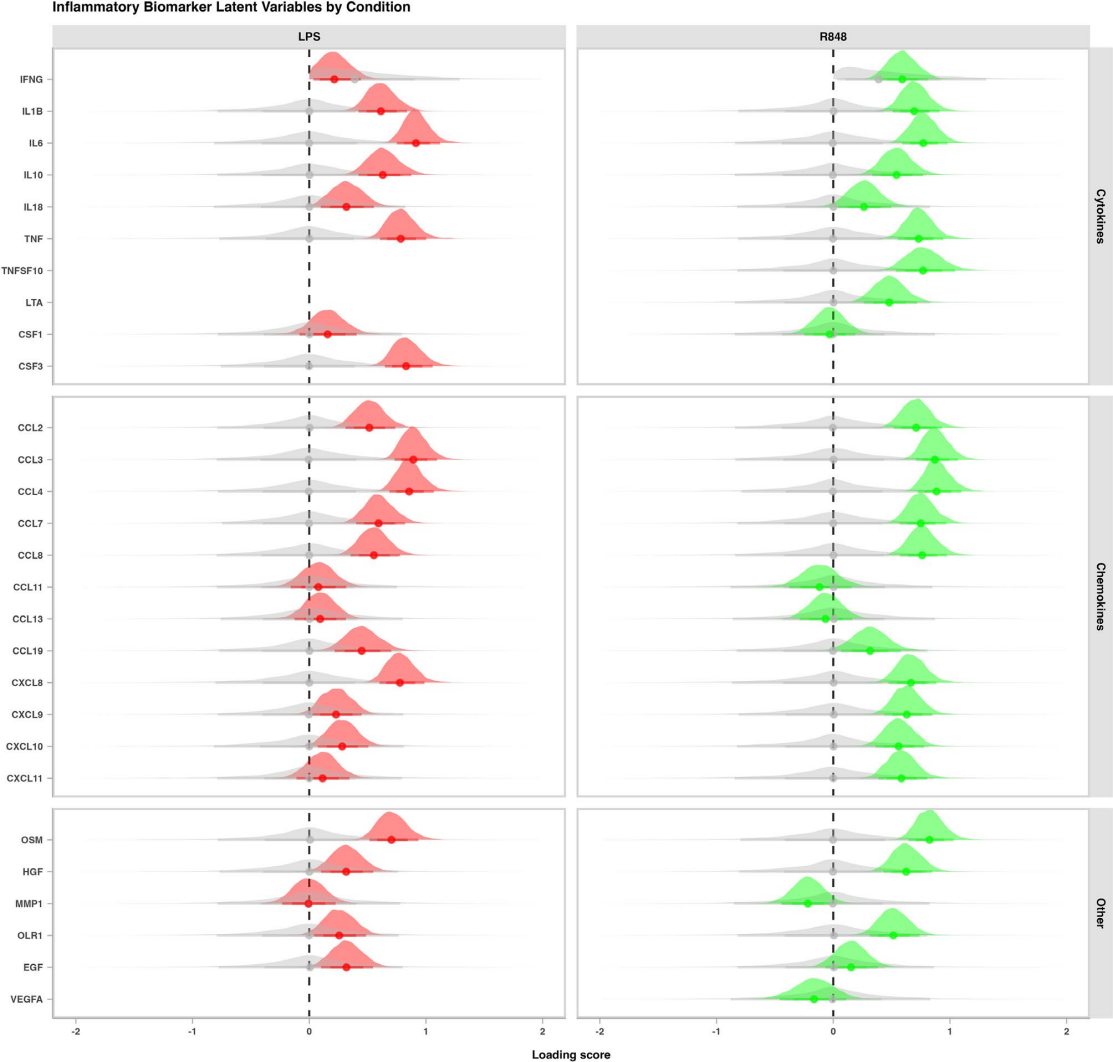
Latent variables of blood cytokine levels in response to lipopolysaccharide and resiquimod stimulation. Posterior densities of latent variable loadings for cytokines in all athletes (N = 52) following 24 h stimulation with lipopolysaccharide (LPS, 100 ng/mL) (left panel, red), or resiquimod (R848, 1uM) (right panel, green). The x axis shows the posterior correlation of each cytokine to the latent variable in each stimulation condition, while the grey density plots represent the prior distributions. Density plots were derived from 6000 posterior draws, with dots representing the mean of the posterior densities, and the thick and thin lines representing the 70% and 90% intervals, respectively

Two latent variables were derived from stimulated cytokine values: a latent variable of LPS reactivity, and a latent variable of R848 reactivity. The posterior estimates of the cytokine/chemokine correlations to the latent structure for each model can be seen in Fig. 2. As expected, cytokines Interleukin (IL)-6, tumor necrosis factor (TNF)-α, colony stimulating factor (CSF)-3, and chemokine ligands (CCLs)-3,4, and C-X-C motif chemokine ligand (CXCL)-8, loaded highly on the LPS latent variable, as these are known to be released in response to LPS through the TLR4/Nuclear Factor Kappa B (NF-κB) pathway [54]. Also as expected, the R848 latent variable had many similar important cytokine loadings [55], but differed slightly from LPS by inducing a greater chemokine response. Latent modelling for both R848 and LPS stimulated conditions was completed on all 52 samples.

### Preliminary evidence of an effect of SRC on immune function

**Fig. 3:**
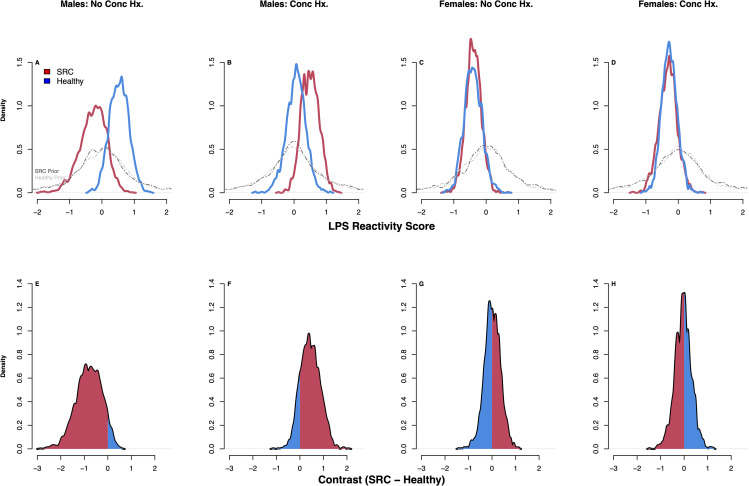
Posterior densities derived from student-t modelling of LPS reactivity. SRC, sport-related concussion; Conc Hx, concussion history; LPS, lipopolysaccharide. Density plots show the posterior distributions for the latent cytokine variable of LPS reactivity across groups (SRC, red; Healthy, blue) (**A**–**D**). The grey lines represent the prior distributions for each group in each comparison. Panels **E–H** show the contrasts (difference in SRC – Healthy) for each of the 4 comparisons: The amount of posterior mass to the right of zero indicates the posterior probability that LPS reactivity is higher in SRC, while the amount of posterior mass to the left of zero indicates the posterior probability that LPS reactivity is lower in SRC. The red shading indicates which side of zero has more of the probability mass. For example, in panel **E**, most of the posterior mass is below zero, indicating that LPS reactivity is lower in athletes with SRC, while panel **F** has most of its posterior mass above zero, indicating that LPS reactivity is higher in SRC; these plots coincide with the distributions in **A & B**, respectively. Panels **G** & **H** are equivocal, and coincide with the distributions in **C & D**, respectively. Plots were derived from 2000 posterior draws

**Fig. 4:**
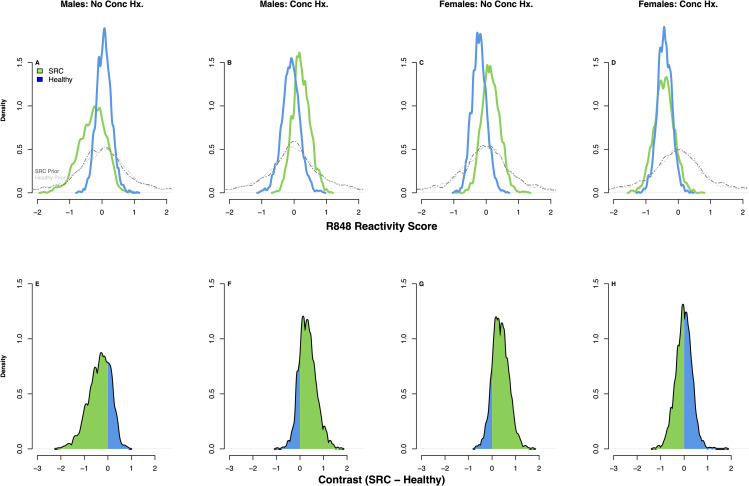
Posterior densities derived from student-t modelling of R848 reactivity. SRC, sport-related concussion; Conc Hx, concussion history; R848, resiquimod. Density plots show the posterior distributions for the latent cytokine variable of R848 reactivity across groups (SRC, green; Healthy, blue) (**A**–**D**). The grey lines represent the prior distributions for each group in each comparison. Panels **E**–**H** show the contrasts (difference in SRC – Healthy) for each of the 4 comparisons: The amount of posterior mass to the right of zero indicates the posterior probability that R848 reactivity is higher in SRC, while the amount of posterior mass to the left of zero indicates the posterior probability that R848 reactivity is lower in SRC. The green shading indicates which side of zero has more of the probability mass. For example, in panel **E**, most of the posterior mass is below zero, indicating that R848 reactivity is lower in athletes with SRC, while panel **F** has most of its posterior mass above zero, indicating that R848 reactivity is higher in SRC; these plots coincide with the distributions in **A & B**, respectively. Panel **G** has most of its posterior mass above zero, indicating that R848 reactivity is higher in SRC, while Panel **H** is equivocal; these plots coincide with the distributions in **C & D**, respectively. Plots were derived from 2000 posterior draws

Student-t derived posterior estimates of the differences (contrasts) in LPS reactivity and R848 reactivity between athletes with SRC and healthy athletes under the modelling assumptions of our DAG (Fig. 1) can be seen in the density plots shown in Figs. 3 and 4. In males with no history of SRC, those with an acute SRC (n = 3) had lower LPS reactivity compared to healthy athletes (n = 8) with 93% posterior probability (pprob) (estimated mean difference (emd) = -0.82 SD units, 90% compatibility interval [CI] -1.15–0.3 SD units); they also had slightly reduced R848 reactivity with 77% pprob (emd = -0.35 SD units, 90% CI = -0.23–0.91 SD units). Conversely, in males with a history of SRC, those with an acute SRC (n = 8) had higher LPS reactivity compared to healthy athletes (n = 4) with 85% pprob (emd = 0.45 SD units, 90% CI -0.16–1.14 SD units), and higher R848 reactivity with 82% pprob (emd = -0.35 SD units, 90% CI = -1.15–0.3 SD units). In females, irrespective of concussion history, there was no effect of SRC on LPS reactivity. However, in females with no concussion history, those with an acute SRC (n = 7) had higher R848 reactivity compared to healthy athletes (n = 10) with 86% pprob (90% CI = -0.18–0.92 SD units).

## Discussion

In this preliminary study, we utilized *ex*-*vivo* whole blood stimulation with known cytokine-producing inflammatory agents to better approximate immune function in individuals following SRC. To foster transparency and reproducibility, we made all statistical modelling assumptions explicit using a causal framework in the form of a DAG. Our DAG was constructed on both our own prior work in the space, as well as others. Our *a priori* heuristic model suggested that SRC would influence immune function, that the effect would be different in males and females, and may be influenced by a prior concussion history. The results of our initial modelling suggest that the effect of an acute SRC on males depends on their concussion history; those with no history of concussion appear to have lower immune reactivity while those with a concussion history appear to have greater immune reactivity compared to their respective healthy counterparts. This effect was not present in females, although there was evidence that females with no concussion history may have increased reactivity to R848 following SRC.

The immune priming hypothesis discovered in animal models of TBI suggests that microglial cells ‘activated’ from a prior injury may overreact to a subsequent injury [33, 38, 56]. This process may then compound with successive insults over time, leading to aberrant inflammatory signaling in the brain that may cause/expedite neurodegeneration [32, 33]. A primed microglia is defined by (1) a higher baseline level of inflammatory mediators, (2) a lower threshold for activation, and (3) an exaggerated response following activation [33]. We found that males with an acute SRC and with a history of concussion had an elevated cytokine response to stimulation with both LPS and R848 compared to their healthy counterparts, suggests a potentially overactive or ‘inflamed’ state. If we were to map the priming definition to our proxy of systemic immune function, we found evidence of (3) an exaggerated response following activation – in males. However, we were unable to test (1) and (2), because we did not measure baseline mediators to assess the former, and the current study was not designed to measure the latter. We are encouraged by these findings, and believe the priming hypothesis warrants further investigation in humans.

We observed that males with an acute SRC and no history of concussion had comparatively lower stimulated cytokine levels to their healthy counterparts in response to both LPS and R848, suggesting possible immunosuppression. Downregulated inflammatory genes have been observed previously in the days following SRC [16], although functional interpretation of static gene expression is difficult. For example, IL-6 can be both pro- and anti-inflammatory given the context [57], and even then, that a known proinflammatory marker like TNF-α is elevated in the blood doesn’t necessarily reflect the current state of the immune system – it may reflect current activity, or it may reflect a recently-active system that is now anergic and suppressed. In the current study, we attempted to make interpretation more intuitive by approximating the current function and state of the immune system through stimulation. The results of our study suggest that male athletes with their first SRC may be immunosuppressed, but validation on a larger sample is needed.

We found an elevated cytokine/chemokine response to R848 stimulation following SRC in females with no concussion history – the opposite of what we found in males with no history of concussion. Of importance, the results reiterate our prior work on sex differences in cytokine signatures following SRC [9], and further supports the need to evaluate males and females separately following injury, particularly when looking at their biology. It is unclear why we observed these sex-disparate findings, although they are wholly unsurprising given the differences in male and female immune function generally [58–60]; indeed, we found that healthy female athletes had a lower cytokine response to LPS compared to healthy male athletes with 86% pprob. However, given the small sample size, and that we found R848 but not LPS reactivity to be altered following SRC in females despite the significant overlap in transcription factor activation between the two stimulants, we caution that further investigation is warranted before these initial findings are generalized.

### Limitations and future directions

We refer to the findings of this study as preliminary because of the limited sample size, relative simplicity of our DAG, and reliance on linear models. The adjustments for sex and concussion history required to estimate the total causal effect of SRC on immune function yielded small effective sample sizes for estimation of the interaction term parameters. However, regularizing priors and pooling of the interaction term helped strengthened the estimates in these low coverage spaces [22], and out-of-sample testing revealed no leverage points. The simplicity of the DAG in Fig. 1 was intentional, in that we wanted to provide an intuitive example of how causal modelling can be used in the SRC biomarker space to estimate causal effects. Beyond the unmeasured effect of exercise, we acknowledge there are many other possible additions/modifications to our causal model, and we hope that our colleagues build upon this in future studies. For example, the role of sex on immune function in this model may be further nuanced by the implications of the female menstrual cycle. Collision sport participation and exposure to repeated head contact may also interact with an acute concussion similarly to concussion history in our model. Genetic variability, presence of comorbid mental health disorders, time from injury to sample acquisition, and many other factors may be added to the DAG in our study or used for the creation of several other DAGs. Because we were explicit in all our assumptions, this will help in the design of future studies regardless of whether they are building upon, replicating, or refuting the findings of this study. Additionally, while we realize that linear models have been useful and intuitive to interpret across much of scientific research, there is no reason to believe that the effects of SRC on immune function are most closely approximated by a line. We believe that there is utility in the simplicity of linear modelling, and that a student-t regression was useful in this sample because of its flexibility in modeling data points in the tails of the distribution. Nonetheless, we encourage future studies to look for non-linear alternatives, including bespoke models, that may better approximate the data generating process. And, finally, it is important to consider that we did not evaluate reactivity of the entire immune system, but rather two specific pathways commonly associated with innate immunity in response to bacterial challenge: the TLR4/NF- κB pathway via LPS, and the TLR7/TLR8 pathway through R848. These two stimulants provided a proxy of the ability of study participants to mount an inflammatory response via two mechanisms that impact a broad suite of cytokines and chemokines. We encourage future studies to continue to look at immune stimulation experiments using different ligands; for example, it would be interesting to know the effects of SRC on viral immunity.

## Conclusion

Whole blood stimulation is a practical and insightful technique that can be used to evaluate immune function post SRC. Moreover, employing an explicit causal framework will facilitate the replication of findings and drive enhancements in subsequent research endeavors. Our preliminary findings indicate that SRC impacts immune function, with a more pronounced effect in male athletes. This effect varies according to concussion history: males without a concussion history tend to exhibit a depressed inflammatory response, while males with a concussion history may have an amplified inflammatory response. Replication of this study in a larger cohort with a more sophisticated causal model is necessary.

### Electronic supplementary material

Below is the link to the electronic supplementary material.

Supplementary Material 1

Supplementary Material 2

Supplementary Material 3

## Data Availability

An altered dataset and code used in the study are available in a GitHub repository located at the following link: https://github.com/dibatti5/Di-Battista-et-al-2023-JNI-Whole-blood-stimulation-.
