# Supplementary material for: Whole blood stimulation provides preliminary evidence of altered immune function following SRC
Source: BMC Immunol. 2024 Jan 13;25:6. doi: 10.1186/s12865-023-00595-8 (PMC10788016; doi:10.1186/s12865-023-00595-8)
Supplement: Supplementary file 1 — Supplementary Material 1 [file 12865_2023_595_MOESM1_ESM.docx]

**Supplementary Methods**

*Bayesian Latent Variable Modelling*

The model was coded with lower triangular loading matrices to conserve identifiability, and employed the method of Leung and Drton [1] to ensure order invariance by inducing priors on the diagonal elements of the loading matrix. Briefly, in a latent factor model, individual biomarker values y arises from a multivariate normal distribution with mean ηBᵀ and covariance matrix Ψ, where Ψ is derived from the factor loadings L. The factor loading matrix is represented by B, and η represents the factor scores, which are distributed as multivariate normal with mean 𝜇, and covariance matrix Σ. The covariance matrix Σ represents the variability in factor scores. The hyperparameter for the loading matrix L is represented by σ_L,_ and the residual error standard deviations are represented by σ_y_. Additionally, β_lower tri_ represents the prior for the loading matrix elements, and β_diag_ represents the prior diagonal entries for the loading matrix. All data were z-score transformed prior to modelling.

y ~ MVNormal(ηBᵀ, Ψ)

Ψ = L * Lᵀ + diag(σ_y_)

η ~ MVNormal(𝜇, Σ)

Σ = L * Lᵀ

σ_L_ ~ Normal(0, 0.5)

σ_y_ ~ Normal(0, 0.5)

β_lower tri_ ~ Normal(0, σ_L_)

Diagonal Element Prior: (k - i) * log(β_diag_[i]) - 0.5 * β_diag_[i]² / σ_L_

[1]

Student-t modelling notation used to predict latent variable scores (y) derived from model [1].

y ~ StudentT(ν, 𝜇, σ)

𝜇 = α_[group (SRC/Healthy)]_ + β_sex_ + γ_[conc Hx]_ + δ_[group*sex*conc Hx]_

α,β,γ ~ Normal(0, 0.2)

δ ~ Normal(𝜇_δ, σ_δ)

𝜇_δ ~ Normal(0,0.2)

σ, σ_δ ~ Exponential(1)

ν ~ gamma(2,0.1)

[2]

**References**

1. Leung D, Drton M. Order-invariant prior specification in Bayesian factor analysis. Statistics & Probability Letters. 2016;111:60–6.
