# Supplementary material for: Whole blood stimulation provides preliminary evidence of altered immune function following SRC
Source: BMC Immunol. 2024 Jan 13;25:6. doi: 10.1186/s12865-023-00595-8 (PMC10788016; doi:10.1186/s12865-023-00595-8)
Supplement: Supplementary file 2 — Supplementary Material 2 [file 12865_2023_595_MOESM2_ESM.docx]

| **Supplementary Table 1.** Raw Biomarker Values | | | | |
| --- | --- | --- | --- | --- |
| **Biomarker** | **LPS** | | **R848** | |
|  | **Healthy**, N = 30*^1^* | **SRC**, N = 22*^1^* | **Healthy**, N = 30*^1^* | **SRC**, N = 22*^1^* |
| **IFNG** | 1,892.4 (860.6 – 2,872.4) | 1,589.2 (916.2 – 3,252.3) | 929.6 (530.9 – 1,653.5) | 1,437.7 (1,008.3 – 2,886.8) |
| **IL1B** | 6,467.5 (4,133.0 – 7,732.4) | 4,669.7 (3,264.4 – 6,076.6) | 430.8 (320.7 – 601.9) | 552.8 (290.4 – 844.7) |
| **IL2** | NA (NA – NA) | NA (NA – NA) | NA (NA – NA) | NA (NA – NA) |
| **IL4** | NA (NA – NA) | NA (NA – NA) | NA (NA – NA) | NA (NA – NA) |
| **IL6** | 48,266.9 (36,709.9 – 58,035.4) | 47,978.7 (40,821.8 – 53,614.0) | 9,528.3 (7,331.1 – 11,798.7) | 9,162.5 (7,657.9 – 13,095.7) |
| **IL7** | NA (NA – NA) | NA (NA – NA) | NA (NA – NA) | NA (NA – NA) |
| **IL10** | 88.9 (71.2 – 138.3) | 80.5 (60.2 – 118.8) | 219.3 (166.3 – 264.2) | 210.6 (177.0 – 285.7) |
| **IL13** | NA (NA – NA) | NA (NA – NA) | NA (NA – NA) | NA (NA – NA) |
| **IL15** | 20.3 (15.3 – 27.4) | 24.7 (21.0 – 42.0) | 33.5 (25.5 – 41.4) | 19.3 (16.0 – 22.6) |
| **IL17A** | 14.9 (12.7 – 26.6) | 14.6 (13.5 – 16.2) | 14.0 (12.6 – 15.8) | 16.1 (15.1 – 16.7) |
| **IL17C** | NA (NA – NA) | NA (NA – NA) | NA (NA – NA) | NA (NA – NA) |
| **IL17F** | NA (NA – NA) | 118.9 (118.9 – 118.9) | NA (NA – NA) | 143.2 (143.2 – 143.2) |
| **IL18** | 420.8 (273.9 – 474.5) | 368.3 (333.9 – 492.2) | 311.8 (245.0 – 425.5) | 395.7 (331.1 – 492.8) |
| **IL27** | NA (NA – NA) | NA (NA – NA) | NA (NA – NA) | NA (NA – NA) |
| **IL33** | NA (NA – NA) | NA (NA – NA) | NA (NA – NA) | NA (NA – NA) |
| **TNF** | 12,276.6 (9,778.8 – 15,201.1) | 12,103.1 (8,917.7 – 14,143.3) | 5,880.7 (4,846.1 – 6,829.5) | 4,844.5 (3,905.3 – 7,247.0) |
| **TNFSF10** | 151.8 (140.4 – 163.2) | 111.0 (105.7 – 137.0) | 112.7 (107.1 – 143.4) | 119.4 (108.1 – 131.2) |
| **TNFSF12** | NA (NA – NA) | NA (NA – NA) | NA (NA – NA) | NA (NA – NA) |
| **FLT3LG** | NA (NA – NA) | NA (NA – NA) | NA (NA – NA) | NA (NA – NA) |
| **LTA** | 12.8 (12.4 – 13.1) | 19.4 (16.6 – 22.2) | 24.6 (20.6 – 33.2) | 26.9 (23.1 – 32.9) |
| **CSF1** | 34.0 (32.3 – 38.2) | 32.6 (29.9 – 34.5) | 34.1 (29.4 – 39.9) | 33.8 (30.7 – 37.8) |
| **CSF2** | 39.3 (32.6 – 47.9) | 32.6 (30.2 – 33.7) | 28.7 (28.5 – 37.3) | 41.1 (27.5 – 49.2) |
| **CSF3** | 2,127.5 (1,726.9 – 2,809.4) | 1,952.1 (1,436.8 – 2,550.3) | NA (NA – NA) | NA (NA – NA) |
| **OSM** | 115.8 (81.6 – 165.5) | 112.7 (96.1 – 153.4) | 24.2 (16.8 – 34.7) | 24.8 (22.3 – 35.4) |
| **TSLP** | NA (NA – NA) | NA (NA – NA) | NA (NA – NA) | NA (NA – NA) |
| **CCL2** | 4,507.3 (2,894.2 – 5,766.8) | 3,647.5 (3,115.6 – 4,723.2) | 14,306.6 (10,945.2 – 19,244.0) | 14,332.4 (12,079.6 – 19,462.2) |
| **CCL3** | 29,033.6 (23,105.4 – 44,714.2) | 30,903.0 (27,181.5 – 40,455.8) | 11,706.0 (8,905.9 – 13,360.7) | 12,547.9 (10,142.7 – 16,298.4) |
| **CCL4** | 69,883.7 (56,304.2 – 114,789.4) | 84,043.1 (61,602.1 – 98,782.8) | 39,765.7 (32,110.2 – 54,129.5) | 43,745.6 (32,184.3 – 55,678.0) |
| **CCL7** | 476.6 (374.3 – 617.8) | 382.0 (312.9 – 430.5) | 500.8 (380.2 – 601.6) | 471.9 (390.6 – 645.1) |
| **CCL8** | 940.8 (688.9 – 1,874.4) | 919.0 (795.6 – 1,224.9) | 4,296.0 (3,282.4 – 6,068.4) | 4,546.1 (3,598.3 – 5,879.5) |
| **CCL11** | 94.3 (74.6 – 112.4) | 101.9 (81.3 – 128.3) | 98.4 (80.5 – 117.0) | 118.9 (90.4 – 143.5) |
| **CCL13** | 63.2 (49.8 – 89.5) | 89.9 (64.6 – 112.0) | 66.7 (52.6 – 87.5) | 100.3 (68.1 – 117.1) |
| **CCL19** | 173.1 (122.1 – 222.4) | 153.7 (124.2 – 168.5) | 131.3 (117.0 – 186.2) | 115.5 (108.0 – 122.1) |
| **CXCL8** | 12,868.9 (10,533.2 – 17,678.9) | 13,913.4 (10,255.0 – 15,903.7) | 513.9 (422.2 – 664.5) | 654.7 (496.9 – 1,109.2) |
| **CXCL9** | 253.0 (179.0 – 452.4) | 291.0 (165.8 – 396.2) | 175.5 (136.0 – 261.6) | 213.2 (142.4 – 334.9) |
| **CXCL10** | 3,520.1 (2,229.4 – 5,386.5) | 4,221.9 (2,536.5 – 4,623.6) | 18,466.9 (14,589.8 – 23,854.5) | 16,861.3 (13,377.6 – 22,257.0) |
| **CXCL11** | 448.9 (337.4 – 562.0) | 539.1 (417.0 – 845.3) | 1,001.6 (823.3 – 1,273.0) | 1,159.3 (899.7 – 1,481.4) |
| **CXCL12** | NA (NA – NA) | NA (NA – NA) | NA (NA – NA) | NA (NA – NA) |
| **HGF** | 110.3 (95.5 – 155.0) | 149.1 (109.0 – 167.1) | 97.5 (70.7 – 116.5) | 123.2 (91.3 – 144.4) |
| **MMP1** | 1,107.6 (773.9 – 1,662.7) | 993.0 (764.0 – 1,465.7) | 1,087.0 (738.2 – 1,523.7) | 1,049.9 (673.9 – 1,731.0) |
| **MMP12** | NA (NA – NA) | NA (NA – NA) | NA (NA – NA) | NA (NA – NA) |
| **OLR1** | 237.1 (147.0 – 314.6) | 300.2 (129.9 – 452.4) | 178.8 (140.4 – 265.5) | 177.1 (130.4 – 455.4) |
| **EGF** | 214.2 (188.4 – 249.1) | 238.3 (191.5 – 265.2) | 224.7 (193.8 – 255.8) | 240.0 (203.1 – 287.6) |
| **TGFA** | NA (NA – NA) | NA (NA – NA) | NA (NA – NA) | NA (NA – NA) |
| **VEGFA** | 58.9 (52.7 – 64.7) | 55.9 (49.4 – 59.7) | 59.8 (50.0 – 72.0) | 62.8 (58.4 – 66.8) |
| *^1^* Median (IQR)  LPS, lipopolysaccharide; R848, resiquimod; SRC, sport-related concussion. | | | | |
