# Supplementary material for: Whole blood stimulation provides preliminary evidence of altered immune function following SRC
Source: BMC Immunol. 2024 Jan 13;25:6. doi: 10.1186/s12865-023-00595-8 (PMC10788016; doi:10.1186/s12865-023-00595-8)
Supplement: Supplementary file 3 — Supplementary Material 3 [file 12865_2023_595_MOESM3_ESM.docx]

**Supplementary Table 2.** Biomarker Quantitation Data

| **Biomarker** | **LPS** | | **R848** | |  |
| --- | --- | --- | --- | --- | --- |
|  | **SRC**, N = 22*^1^* | **Healthy**, N = 30*^1^* | **SRC**, N = 22*^1^* | **Healthy**, N = 30*^1^* | **SRC**, N = 22*^1^* |
| **CCL8** |  |  |  |  |  |
| Within LOQ | 22 (100) | 30 (100) | 22 (100) | 30 (100) | 22 (100) |
| Above ULOQ | 0 (0) | 0 (0) | 0 (0) | 0 (0) | 0 (0) |
| Below LLOQ | 0 (0) | 0 (0) | 0 (0) | 0 (0) | 0 (0) |
| Below LLOD | 0 (0) | 0 (0) | 0 (0) | 0 (0) | 0 (0) |
| MCAR | 0 (0) | 0 (0) | 0 (0) | 0 (0) | 0 (0) |
| **IL33** |  |  |  |  |  |
| Within LOQ | 0 (0) | 0 (0) | 0 (0) | 0 (0) | 0 (0) |
| Above ULOQ | 0 (0) | 0 (0) | 0 (0) | 0 (0) | 0 (0) |
| Below LLOQ | 20 (91) | 0 (0) | 1 (4.5) | 1 (3.3) | 0 (0) |
| Below LLOD | 2 (9.1) | 30 (100) | 21 (95) | 29 (97) | 22 (100) |
| MCAR | 0 (0) | 0 (0) | 0 (0) | 0 (0) | 0 (0) |
| **CXCL12** |  |  |  |  |  |
| Within LOQ | 18 (82) | 0 (0) | 0 (0) | 0 (0) | 0 (0) |
| Above ULOQ | 0 (0) | 0 (0) | 0 (0) | 0 (0) | 0 (0) |
| Below LLOQ | 4 (18) | 12 (40) | 14 (64) | 12 (40) | 8 (36) |
| Below LLOD | 0 (0) | 18 (60) | 8 (36) | 18 (60) | 14 (64) |
| MCAR | 0 (0) | 0 (0) | 0 (0) | 0 (0) | 0 (0) |
| **OLR1** |  |  |  |  |  |
| Within LOQ | 22 (100) | 26 (87) | 21 (95) | 20 (67) | 18 (82) |
| Above ULOQ | 0 (0) | 0 (0) | 0 (0) | 0 (0) | 0 (0) |
| Below LLOQ | 0 (0) | 4 (13) | 1 (4.5) | 10 (33) | 4 (18) |
| Below LLOD | 0 (0) | 0 (0) | 0 (0) | 0 (0) | 0 (0) |
| MCAR | 0 (0) | 0 (0) | 0 (0) | 0 (0) | 0 (0) |
| **IL27** |  |  |  |  |  |
| Within LOQ | 5 (23) | 0 (0) | 0 (0) | 0 (0) | 0 (0) |
| Above ULOQ | 0 (0) | 0 (0) | 0 (0) | 0 (0) | 0 (0) |
| Below LLOQ | 17 (77) | 30 (100) | 22 (100) | 30 (100) | 22 (100) |
| Below LLOD | 0 (0) | 0 (0) | 0 (0) | 0 (0) | 0 (0) |
| MCAR | 0 (0) | 0 (0) | 0 (0) | 0 (0) | 0 (0) |
| **IL2** |  |  |  |  |  |
| Within LOQ | 0 (0) | 0 (0) | 0 (0) | 0 (0) | 0 (0) |
| Above ULOQ | 0 (0) | 0 (0) | 0 (0) | 0 (0) | 0 (0) |
| Below LLOQ | 20 (91) | 27 (90) | 20 (91) | 25 (83) | 21 (95) |
| Below LLOD | 0 (0) | 2 (6.7) | 1 (4.5) | 4 (13) | 0 (0) |
| MCAR | 2 (9.1) | 1 (3.3) | 1 (4.5) | 1 (3.3) | 1 (4.5) |
| **CXCL9** |  |  |  |  |  |
| Within LOQ | 22 (100) | 30 (100) | 22 (100) | 30 (100) | 22 (100) |
| Above ULOQ | 0 (0) | 0 (0) | 0 (0) | 0 (0) | 0 (0) |
| Below LLOQ | 0 (0) | 0 (0) | 0 (0) | 0 (0) | 0 (0) |
| Below LLOD | 0 (0) | 0 (0) | 0 (0) | 0 (0) | 0 (0) |
| MCAR | 0 (0) | 0 (0) | 0 (0) | 0 (0) | 0 (0) |
| **TGFA** |  |  |  |  |  |
| Within LOQ | 17 (77) | 0 (0) | 0 (0) | 0 (0) | 0 (0) |
| Above ULOQ | 0 (0) | 0 (0) | 0 (0) | 0 (0) | 0 (0) |
| Below LLOQ | 5 (23) | 0 (0) | 0 (0) | 0 (0) | 0 (0) |
| Below LLOD | 0 (0) | 30 (100) | 22 (100) | 30 (100) | 22 (100) |
| MCAR | 0 (0) | 0 (0) | 0 (0) | 0 (0) | 0 (0) |
| **IL1B** |  |  |  |  |  |
| Within LOQ | 15 (68) | 30 (100) | 22 (100) | 30 (100) | 22 (100) |
| Above ULOQ | 0 (0) | 0 (0) | 0 (0) | 0 (0) | 0 (0) |
| Below LLOQ | 7 (32) | 0 (0) | 0 (0) | 0 (0) | 0 (0) |
| Below LLOD | 0 (0) | 0 (0) | 0 (0) | 0 (0) | 0 (0) |
| MCAR | 0 (0) | 0 (0) | 0 (0) | 0 (0) | 0 (0) |
| **IL6** |  |  |  |  |  |
| Within LOQ | 21 (95) | 30 (100) | 22 (100) | 30 (100) | 22 (100) |
| Above ULOQ | 1 (4.5) | 0 (0) | 0 (0) | 0 (0) | 0 (0) |
| Below LLOQ | 0 (0) | 0 (0) | 0 (0) | 0 (0) | 0 (0) |
| Below LLOD | 0 (0) | 0 (0) | 0 (0) | 0 (0) | 0 (0) |
| MCAR | 0 (0) | 0 (0) | 0 (0) | 0 (0) | 0 (0) |
| **IL4** |  |  |  |  |  |
| Within LOQ | 2 (9.1) | 0 (0) | 0 (0) | 0 (0) | 0 (0) |
| Above ULOQ | 0 (0) | 0 (0) | 0 (0) | 0 (0) | 0 (0) |
| Below LLOQ | 20 (91) | 28 (93) | 18 (82) | 24 (80) | 16 (73) |
| Below LLOD | 0 (0) | 0 (0) | 0 (0) | 0 (0) | 0 (0) |
| MCAR | 0 (0) | 2 (6.7) | 4 (18) | 6 (20) | 6 (27) |
| **TNFSF12** |  |  |  |  |  |
| Within LOQ | 22 (100) | 0 (0) | 0 (0) | 0 (0) | 0 (0) |
| Above ULOQ | 0 (0) | 0 (0) | 0 (0) | 0 (0) | 0 (0) |
| Below LLOQ | 0 (0) | 30 (100) | 22 (100) | 30 (100) | 22 (100) |
| Below LLOD | 0 (0) | 0 (0) | 0 (0) | 0 (0) | 0 (0) |
| MCAR | 0 (0) | 0 (0) | 0 (0) | 0 (0) | 0 (0) |
| **TSLP** |  |  |  |  |  |
| Within LOQ | 1 (4.5) | 0 (0) | 0 (0) | 0 (0) | 0 (0) |
| Above ULOQ | 0 (0) | 0 (0) | 0 (0) | 0 (0) | 0 (0) |
| Below LLOQ | 4 (18) | 1 (3.3) | 1 (4.5) | 1 (3.3) | 0 (0) |
| Below LLOD | 17 (77) | 29 (97) | 21 (95) | 29 (97) | 22 (100) |
| MCAR | 0 (0) | 0 (0) | 0 (0) | 0 (0) | 0 (0) |
| **CCL11** |  |  |  |  |  |
| Within LOQ | 22 (100) | 28 (93) | 20 (91) | 28 (93) | 20 (91) |
| Above ULOQ | 0 (0) | 0 (0) | 0 (0) | 0 (0) | 0 (0) |
| Below LLOQ | 0 (0) | 2 (6.7) | 2 (9.1) | 2 (6.7) | 2 (9.1) |
| Below LLOD | 0 (0) | 0 (0) | 0 (0) | 0 (0) | 0 (0) |
| MCAR | 0 (0) | 0 (0) | 0 (0) | 0 (0) | 0 (0) |
| **HGF** |  |  |  |  |  |
| Within LOQ | 22 (100) | 30 (100) | 22 (100) | 30 (100) | 22 (100) |
| Above ULOQ | 0 (0) | 0 (0) | 0 (0) | 0 (0) | 0 (0) |
| Below LLOQ | 0 (0) | 0 (0) | 0 (0) | 0 (0) | 0 (0) |
| Below LLOD | 0 (0) | 0 (0) | 0 (0) | 0 (0) | 0 (0) |
| MCAR | 0 (0) | 0 (0) | 0 (0) | 0 (0) | 0 (0) |
| **FLT3LG** |  |  |  |  |  |
| Within LOQ | 22 (100) | 0 (0) | 0 (0) | 0 (0) | 0 (0) |
| Above ULOQ | 0 (0) | 0 (0) | 0 (0) | 0 (0) | 0 (0) |
| Below LLOQ | 0 (0) | 30 (100) | 22 (100) | 30 (100) | 22 (100) |
| Below LLOD | 0 (0) | 0 (0) | 0 (0) | 0 (0) | 0 (0) |
| MCAR | 0 (0) | 0 (0) | 0 (0) | 0 (0) | 0 (0) |
| **IL17F** |  |  |  |  |  |
| Within LOQ | 2 (9.1) | 0 (0) | 1 (4.5) | 0 (0) | 1 (4.5) |
| Above ULOQ | 0 (0) | 0 (0) | 0 (0) | 0 (0) | 0 (0) |
| Below LLOQ | 19 (86) | 30 (100) | 20 (91) | 29 (97) | 21 (95) |
| Below LLOD | 1 (4.5) | 0 (0) | 1 (4.5) | 1 (3.3) | 0 (0) |
| MCAR | 0 (0) | 0 (0) | 0 (0) | 0 (0) | 0 (0) |
| **IL7** |  |  |  |  |  |
| Within LOQ | 22 (100) | 0 (0) | 0 (0) | 0 (0) | 0 (0) |
| Above ULOQ | 0 (0) | 0 (0) | 0 (0) | 0 (0) | 0 (0) |
| Below LLOQ | 0 (0) | 30 (100) | 22 (100) | 30 (100) | 22 (100) |
| Below LLOD | 0 (0) | 0 (0) | 0 (0) | 0 (0) | 0 (0) |
| MCAR | 0 (0) | 0 (0) | 0 (0) | 0 (0) | 0 (0) |
| **IL13** |  |  |  |  |  |
| Within LOQ | 1 (4.5) | 0 (0) | 0 (0) | 0 (0) | 0 (0) |
| Above ULOQ | 0 (0) | 0 (0) | 0 (0) | 0 (0) | 0 (0) |
| Below LLOQ | 16 (73) | 7 (23) | 3 (14) | 4 (13) | 2 (9.1) |
| Below LLOD | 5 (23) | 23 (77) | 19 (86) | 26 (87) | 20 (91) |
| MCAR | 0 (0) | 0 (0) | 0 (0) | 0 (0) | 0 (0) |
| **IL18** |  |  |  |  |  |
| Within LOQ | 22 (100) | 30 (100) | 22 (100) | 30 (100) | 22 (100) |
| Above ULOQ | 0 (0) | 0 (0) | 0 (0) | 0 (0) | 0 (0) |
| Below LLOQ | 0 (0) | 0 (0) | 0 (0) | 0 (0) | 0 (0) |
| Below LLOD | 0 (0) | 0 (0) | 0 (0) | 0 (0) | 0 (0) |
| MCAR | 0 (0) | 0 (0) | 0 (0) | 0 (0) | 0 (0) |
| **CCL13** |  |  |  |  |  |
| Within LOQ | 22 (100) | 30 (100) | 22 (100) | 30 (100) | 22 (100) |
| Above ULOQ | 0 (0) | 0 (0) | 0 (0) | 0 (0) | 0 (0) |
| Below LLOQ | 0 (0) | 0 (0) | 0 (0) | 0 (0) | 0 (0) |
| Below LLOD | 0 (0) | 0 (0) | 0 (0) | 0 (0) | 0 (0) |
| MCAR | 0 (0) | 0 (0) | 0 (0) | 0 (0) | 0 (0) |
| **TNFSF10** |  |  |  |  |  |
| Within LOQ | 22 (100) | 2 (6.7) | 6 (27) | 14 (47) | 16 (73) |
| Above ULOQ | 0 (0) | 0 (0) | 0 (0) | 0 (0) | 0 (0) |
| Below LLOQ | 0 (0) | 28 (93) | 16 (73) | 16 (53) | 6 (27) |
| Below LLOD | 0 (0) | 0 (0) | 0 (0) | 0 (0) | 0 (0) |
| MCAR | 0 (0) | 0 (0) | 0 (0) | 0 (0) | 0 (0) |
| **CXCL10** |  |  |  |  |  |
| Within LOQ | 21 (95) | 30 (100) | 22 (100) | 30 (100) | 22 (100) |
| Above ULOQ | 1 (4.5) | 0 (0) | 0 (0) | 0 (0) | 0 (0) |
| Below LLOQ | 0 (0) | 0 (0) | 0 (0) | 0 (0) | 0 (0) |
| Below LLOD | 0 (0) | 0 (0) | 0 (0) | 0 (0) | 0 (0) |
| MCAR | 0 (0) | 0 (0) | 0 (0) | 0 (0) | 0 (0) |
| **IFNG** |  |  |  |  |  |
| Within LOQ | 21 (95) | 30 (100) | 21 (95) | 30 (100) | 21 (95) |
| Above ULOQ | 0 (0) | 0 (0) | 0 (0) | 0 (0) | 0 (0) |
| Below LLOQ | 1 (4.5) | 0 (0) | 1 (4.5) | 0 (0) | 1 (4.5) |
| Below LLOD | 0 (0) | 0 (0) | 0 (0) | 0 (0) | 0 (0) |
| MCAR | 0 (0) | 0 (0) | 0 (0) | 0 (0) | 0 (0) |
| **IL10** |  |  |  |  |  |
| Within LOQ | 5 (23) | 28 (93) | 21 (95) | 30 (100) | 21 (95) |
| Above ULOQ | 0 (0) | 0 (0) | 0 (0) | 0 (0) | 0 (0) |
| Below LLOQ | 17 (77) | 2 (6.7) | 1 (4.5) | 0 (0) | 1 (4.5) |
| Below LLOD | 0 (0) | 0 (0) | 0 (0) | 0 (0) | 0 (0) |
| MCAR | 0 (0) | 0 (0) | 0 (0) | 0 (0) | 0 (0) |
| **CCL19** |  |  |  |  |  |
| Within LOQ | 22 (100) | 23 (77) | 19 (86) | 19 (63) | 10 (45) |
| Above ULOQ | 0 (0) | 0 (0) | 0 (0) | 0 (0) | 0 (0) |
| Below LLOQ | 0 (0) | 7 (23) | 3 (14) | 11 (37) | 12 (55) |
| Below LLOD | 0 (0) | 0 (0) | 0 (0) | 0 (0) | 0 (0) |
| MCAR | 0 (0) | 0 (0) | 0 (0) | 0 (0) | 0 (0) |
| **TNF** |  |  |  |  |  |
| Within LOQ | 19 (86) | 30 (100) | 22 (100) | 30 (100) | 22 (100) |
| Above ULOQ | 0 (0) | 0 (0) | 0 (0) | 0 (0) | 0 (0) |
| Below LLOQ | 3 (14) | 0 (0) | 0 (0) | 0 (0) | 0 (0) |
| Below LLOD | 0 (0) | 0 (0) | 0 (0) | 0 (0) | 0 (0) |
| MCAR | 0 (0) | 0 (0) | 0 (0) | 0 (0) | 0 (0) |
| **IL15** |  |  |  |  |  |
| Within LOQ | 22 (100) | 4 (13) | 3 (14) | 2 (6.7) | 3 (14) |
| Above ULOQ | 0 (0) | 0 (0) | 0 (0) | 0 (0) | 0 (0) |
| Below LLOQ | 0 (0) | 26 (87) | 19 (86) | 28 (93) | 19 (86) |
| Below LLOD | 0 (0) | 0 (0) | 0 (0) | 0 (0) | 0 (0) |
| MCAR | 0 (0) | 0 (0) | 0 (0) | 0 (0) | 0 (0) |
| **CCL3** |  |  |  |  |  |
| Within LOQ | 21 (95) | 30 (100) | 22 (100) | 30 (100) | 22 (100) |
| Above ULOQ | 1 (4.5) | 0 (0) | 0 (0) | 0 (0) | 0 (0) |
| Below LLOQ | 0 (0) | 0 (0) | 0 (0) | 0 (0) | 0 (0) |
| Below LLOD | 0 (0) | 0 (0) | 0 (0) | 0 (0) | 0 (0) |
| MCAR | 0 (0) | 0 (0) | 0 (0) | 0 (0) | 0 (0) |
| **CXCL8** |  |  |  |  |  |
| Within LOQ | 21 (95) | 30 (100) | 22 (100) | 30 (100) | 22 (100) |
| Above ULOQ | 1 (4.5) | 0 (0) | 0 (0) | 0 (0) | 0 (0) |
| Below LLOQ | 0 (0) | 0 (0) | 0 (0) | 0 (0) | 0 (0) |
| Below LLOD | 0 (0) | 0 (0) | 0 (0) | 0 (0) | 0 (0) |
| MCAR | 0 (0) | 0 (0) | 0 (0) | 0 (0) | 0 (0) |
| **MMP12** |  |  |  |  |  |
| Within LOQ | 22 (100) | 0 (0) | 0 (0) | 0 (0) | 0 (0) |
| Above ULOQ | 0 (0) | 0 (0) | 0 (0) | 0 (0) | 0 (0) |
| Below LLOQ | 0 (0) | 10 (33) | 11 (50) | 13 (43) | 15 (68) |
| Below LLOD | 0 (0) | 20 (67) | 11 (50) | 17 (57) | 7 (32) |
| MCAR | 0 (0) | 0 (0) | 0 (0) | 0 (0) | 0 (0) |
| **CSF2** |  |  |  |  |  |
| Within LOQ | 3 (14) | 9 (30) | 5 (23) | 3 (10) | 5 (23) |
| Above ULOQ | 0 (0) | 0 (0) | 0 (0) | 0 (0) | 0 (0) |
| Below LLOQ | 19 (86) | 21 (70) | 16 (73) | 27 (90) | 15 (68) |
| Below LLOD | 0 (0) | 0 (0) | 1 (4.5) | 0 (0) | 2 (9.1) |
| MCAR | 0 (0) | 0 (0) | 0 (0) | 0 (0) | 0 (0) |
| **CSF3** |  |  |  |  |  |
| Within LOQ | 11 (50) | 29 (97) | 20 (91) | 0 (0) | 0 (0) |
| Above ULOQ | 0 (0) | 0 (0) | 0 (0) | 0 (0) | 0 (0) |
| Below LLOQ | 11 (50) | 1 (3.3) | 2 (9.1) | 19 (63) | 17 (77) |
| Below LLOD | 0 (0) | 0 (0) | 0 (0) | 11 (37) | 5 (23) |
| MCAR | 0 (0) | 0 (0) | 0 (0) | 0 (0) | 0 (0) |
| **VEGFA** |  |  |  |  |  |
| Within LOQ | 22 (100) | 10 (33) | 9 (41) | 15 (50) | 12 (55) |
| Above ULOQ | 0 (0) | 0 (0) | 0 (0) | 0 (0) | 0 (0) |
| Below LLOQ | 0 (0) | 20 (67) | 13 (59) | 15 (50) | 10 (45) |
| Below LLOD | 0 (0) | 0 (0) | 0 (0) | 0 (0) | 0 (0) |
| MCAR | 0 (0) | 0 (0) | 0 (0) | 0 (0) | 0 (0) |
| **IL17C** |  |  |  |  |  |
| Within LOQ | 19 (86) | 0 (0) | 0 (0) | 0 (0) | 0 (0) |
| Above ULOQ | 0 (0) | 0 (0) | 0 (0) | 0 (0) | 0 (0) |
| Below LLOQ | 3 (14) | 30 (100) | 22 (100) | 30 (100) | 22 (100) |
| Below LLOD | 0 (0) | 0 (0) | 0 (0) | 0 (0) | 0 (0) |
| MCAR | 0 (0) | 0 (0) | 0 (0) | 0 (0) | 0 (0) |
| **EGF** |  |  |  |  |  |
| Within LOQ | 22 (100) | 30 (100) | 22 (100) | 30 (100) | 22 (100) |
| Above ULOQ | 0 (0) | 0 (0) | 0 (0) | 0 (0) | 0 (0) |
| Below LLOQ | 0 (0) | 0 (0) | 0 (0) | 0 (0) | 0 (0) |
| Below LLOD | 0 (0) | 0 (0) | 0 (0) | 0 (0) | 0 (0) |
| MCAR | 0 (0) | 0 (0) | 0 (0) | 0 (0) | 0 (0) |
| **CCL2** |  |  |  |  |  |
| Within LOQ | 22 (100) | 30 (100) | 22 (100) | 30 (100) | 22 (100) |
| Above ULOQ | 0 (0) | 0 (0) | 0 (0) | 0 (0) | 0 (0) |
| Below LLOQ | 0 (0) | 0 (0) | 0 (0) | 0 (0) | 0 (0) |
| Below LLOD | 0 (0) | 0 (0) | 0 (0) | 0 (0) | 0 (0) |
| MCAR | 0 (0) | 0 (0) | 0 (0) | 0 (0) | 0 (0) |
| **IL17A** |  |  |  |  |  |
| Within LOQ | 3 (14) | 13 (43) | 8 (36) | 9 (30) | 4 (18) |
| Above ULOQ | 0 (0) | 0 (0) | 0 (0) | 0 (0) | 0 (0) |
| Below LLOQ | 18 (82) | 17 (57) | 14 (64) | 21 (70) | 18 (82) |
| Below LLOD | 1 (4.5) | 0 (0) | 0 (0) | 0 (0) | 0 (0) |
| MCAR | 0 (0) | 0 (0) | 0 (0) | 0 (0) | 0 (0) |
| **OSM** |  |  |  |  |  |
| Within LOQ | 22 (100) | 30 (100) | 21 (95) | 28 (93) | 21 (95) |
| Above ULOQ | 0 (0) | 0 (0) | 0 (0) | 0 (0) | 0 (0) |
| Below LLOQ | 0 (0) | 0 (0) | 1 (4.5) | 2 (6.7) | 1 (4.5) |
| Below LLOD | 0 (0) | 0 (0) | 0 (0) | 0 (0) | 0 (0) |
| MCAR | 0 (0) | 0 (0) | 0 (0) | 0 (0) | 0 (0) |
| **CSF1** |  |  |  |  |  |
| Within LOQ | 22 (100) | 28 (93) | 21 (95) | 30 (100) | 22 (100) |
| Above ULOQ | 0 (0) | 0 (0) | 0 (0) | 0 (0) | 0 (0) |
| Below LLOQ | 0 (0) | 2 (6.7) | 1 (4.5) | 0 (0) | 0 (0) |
| Below LLOD | 0 (0) | 0 (0) | 0 (0) | 0 (0) | 0 (0) |
| MCAR | 0 (0) | 0 (0) | 0 (0) | 0 (0) | 0 (0) |
| **CCL4** |  |  |  |  |  |
| Within LOQ | 21 (95) | 30 (100) | 22 (100) | 30 (100) | 22 (100) |
| Above ULOQ | 1 (4.5) | 0 (0) | 0 (0) | 0 (0) | 0 (0) |
| Below LLOQ | 0 (0) | 0 (0) | 0 (0) | 0 (0) | 0 (0) |
| Below LLOD | 0 (0) | 0 (0) | 0 (0) | 0 (0) | 0 (0) |
| MCAR | 0 (0) | 0 (0) | 0 (0) | 0 (0) | 0 (0) |
| **CXCL11** |  |  |  |  |  |
| Within LOQ | 22 (100) | 30 (100) | 22 (100) | 30 (100) | 22 (100) |
| Above ULOQ | 0 (0) | 0 (0) | 0 (0) | 0 (0) | 0 (0) |
| Below LLOQ | 0 (0) | 0 (0) | 0 (0) | 0 (0) | 0 (0) |
| Below LLOD | 0 (0) | 0 (0) | 0 (0) | 0 (0) | 0 (0) |
| MCAR | 0 (0) | 0 (0) | 0 (0) | 0 (0) | 0 (0) |
| **LTA** |  |  |  |  |  |
| Within LOQ | 22 (100) | 2 (6.7) | 2 (9.1) | 29 (97) | 20 (91) |
| Above ULOQ | 0 (0) | 0 (0) | 0 (0) | 0 (0) | 0 (0) |
| Below LLOQ | 0 (0) | 28 (93) | 20 (91) | 1 (3.3) | 2 (9.1) |
| Below LLOD | 0 (0) | 0 (0) | 0 (0) | 0 (0) | 0 (0) |
| MCAR | 0 (0) | 0 (0) | 0 (0) | 0 (0) | 0 (0) |
| **CCL7** |  |  |  |  |  |
| Within LOQ | 22 (100) | 30 (100) | 22 (100) | 30 (100) | 22 (100) |
| Above ULOQ | 0 (0) | 0 (0) | 0 (0) | 0 (0) | 0 (0) |
| Below LLOQ | 0 (0) | 0 (0) | 0 (0) | 0 (0) | 0 (0) |
| Below LLOD | 0 (0) | 0 (0) | 0 (0) | 0 (0) | 0 (0) |
| MCAR | 0 (0) | 0 (0) | 0 (0) | 0 (0) | 0 (0) |
| **MMP1** |  |  |  |  |  |
| Within LOQ | 22 (100) | 30 (100) | 22 (100) | 30 (100) | 22 (100) |
| Above ULOQ | 0 (0) | 0 (0) | 0 (0) | 0 (0) | 0 (0) |
| Below LLOQ | 0 (0) | 0 (0) | 0 (0) | 0 (0) | 0 (0) |
| Below LLOD | 0 (0) | 0 (0) | 0 (0) | 0 (0) | 0 (0) |
| MCAR | 0 (0) | 0 (0) | 0 (0) | 0 (0) | 0 (0) |
| Values presented as the number and percent (%) of samples falling within each category.  LPS, lipopolysaccharide; R848, resiquimod; SRC, sport-related concussion; LOQ, limits of quantitation; ULOQ, upper limit of quantitation; LLOQ, lower limit of quantitation; LLOD, lower limit of detection; MCAR, missing completely at random. | | | | | |
